# Carbamazepine and Diclofenac Removal Double Treatment: Oxidation and Adsorption

**DOI:** 10.3390/ijerph18137163

**Published:** 2021-07-04

**Authors:** Alejandro Aldeguer Esquerdo, Pedro José Varo Galvañ, Irene Sentana Gadea, Daniel Prats Rico

**Affiliations:** University Institute of Water and Environmental Sciences, University of Alicante, 03080 Alicante, Spain; pedro.varo@ua.es (P.J.V.G.); irene.sentana@ua.es (I.S.G.); prats@ua.es (D.P.R.)

**Keywords:** carbamazepine, diclofenac, powdered activated carbon, ozone

## Abstract

In the present research, the effect of two hybrid treatments, ozone followed by powdered activated carbon (PAC) or PAC followed by ozone (O_3_), was studied for the removal of two drugs present in water: diclofenac and carbamazepine. In the study, two initial concentrations of each of the contaminants, 0.7 mg L^−1^ and 1.8 mg L^−1^, were used. Different doses of PAC between 4–20 mg L^−1^ were studied as variables, as well as different doses of O_3_ between 0.056–0.280 mg L^−1^. The evolution of the concentration of each contaminant over time was evaluated. From the results obtained, it was concluded that the combined treatment with ozone followed by PAC reduces between 50% and 75% the time required to achieve 90% removal of diclofenac when compared with the time required when only activated carbon was used. In the case of carbamazepine, the time required was 97% less. For carbamazepine, to achieve reduction percentages of up to 90%, O_3_ treatment followed by PAC acted faster than PAC followed by O_3_. In the case of diclofenac, PAC treatment followed by O_3_ was faster to reach concentrations of up to 90%. However, to reach yields below 80%, O_3_ treatment followed by PAC was more efficient.

## 1. Introduction

Pharmaceuticals are synthetic or natural compounds that are used generally for the treatment of animal or human diseases [1,2]. They are compounds that are characterized by their complex chemical structure meaning that over time they can exert their therapeutic activity, which is why they are characterized by their persistence over time. Several studies confirm the danger of pharmaceutical products towards human health, when present in the environment [3,4].

Carbamazepine (CBZ) is used primarily as an anticonvulsant and mood stabilizer. Its primary use is focused on the treatment of epilepsy and bipolar disorder [5,6]. According to the Biopharmaceutical Classification System (BCS), CBZ is classified as a class II pharmaceutical [7]. According to CBZ’s toxicological properties, it is harmful and dangerous for the aquatic environment and harmful if ingested [8]. Several studies mention the ecotoxicity of CBZ in different aquatic species, with critical effects prevailing in developmental rate or reproduction [9,10]. For example, an average lethal concentration value (LC_50_) of 111 mg L^−1^ after two days of exposure can be found in the crustacean *Daphnia magna* [11]. Mortality effects are also reported for the presence of CBZ in mammals, showing changes in the reduction of male fertility in Wistar rats [12]. Among studies on the possible risks to human health caused by CBZ exposure, the effect on the increase in the development of spina bifida at the intrauterine level stands out [13], along with problems in the neurological development of the fetus [14], increased fetal losses and increased rates of congenital malformation in fetuses [15]. The European Union (EU) included CBZ in the list of substances that need to be studied, identified by The EU Water Framework Directive [16].

The main source of CBZ in surface waters is via effluent from wastewater treatment plants (WWTPs) [17,18], followed by runoff water from heavy rainfall in municipal and hospital solid waste [19,20]. Likewise, CBZ can accumulate in soil from the filtration of irrigation water and by sludge used as fertilizer, from WWTPs [21]. This compound has also been found in seawater due to surface water runoff and groundwater discharge [22], although its concentrations were detected around 2 ng L^−1^ [23]. All of this indicates that CBZ is an extremely persistent compound [24,25].

CBZ concentrations in surface waters are usually higher in dry seasonal periods rather than in wetter ones. CBZ is one of the most detected contaminants in groundwater [26,27]. It has been detected in groundwater in countries such as Japan, USA, Canada, and Germany with concentrations between 1–100 ng L^−1^ [28,29]. It has also been detected in effluents from WWTPs [30,31].

In general, activated sludge treatments (AST) are not effective for reducing contaminants of pharmaceutical origin [32,33]. In contrast, membrane bioreactors (MBRs) achieve higher pharmaceutical reduction efficiencies than ASTs [34,35]. In the case of CBZ, its low reduction efficacy has been reported [35,36] because of its physicochemical properties [37,38]. When both AST and MBR treatments are combined, the reductions achieved range between 5% and 20% [39,40].

The non-mineralization of CBZ in the aquatic systems of WWTPs can form acridine, a toxic by-product when exposed to sunlight [41]. This is why CBZ has been identified as a chemical marker for water contamination [42,43]. It is important to develop effective technologies that allow a greater reduction in pharmaceuticals such as CBZ and solve the current problem that exists with conventional treatments by WWTPs [44].

Diclofenac (DCF) is a molecule with phenylacetic, secondary amine, and phenyl groups with two carbon atoms. Chlorine in the ortho position of the amine causes maximum twisting of the phenyl group, making it a molecule with high polarization. This compound is a non-steroidal anti-inflammatory pharmaceutical (NSAID) with analgesic and anti-inflammatory properties, which is commonly used for the treatment of arthritis [45,46].

Conventional wastewater treatment plants (WWTPs) do not achieve an efficient reduction of most chemical compounds [47], such as pharmaceutical products [48,49]. In the case of DCF, reductions between 21–40% can be achieved [50,51]. By means of AST, various studies confirm reduction rates of DCF that do not reach 50% [52,53]. In turn, ASTs do not achieve complete degradation of DCF, but various metabolites are generated [54]. A membrane bioreactor (MBR) is the most efficient process compared with AST for the reduction of the drug DCF, reaching a reduction of 65% [35]. However, using an anaerobic MBR system, the efficacy of DCF reduction is less than 10% [55].

In Asian countries, the concentration of DCF in wastewater is higher than in European and American countries [56,57]. In general, the concentration of this contaminant is higher in the dry seasons than in the wetter seasons [58]. In addition to wastewater, the presence of DCF has been detected in various bodies of water, both surface and underground [59,60,61,62].

Activated carbon (AC) is a good adsorbent. Each activated carbon has different physicochemical and surface properties that allow them to retain different types of contaminants of emerging concern (CECs), such as pharmaceuticals in water [63,64]. It is a promising technique and several studies confirm this [65,66]. The adsorption capacity of powdered activated carbon (PAC) allows for the efficient reduction of pharmaceuticals present in secondary wastewater [67,68], confirming that it is an effective treatment with economic benefits [69,70].

Advanced oxidation processes (AOP) in wastewater encompass one of the most studied fields of research in recent years, with China being the country that would top the list, due to the increase in contamination of its waters in the last 40 years, caused mainly by vast population growth and industrialization [71], followed by Spain, the USA and India [72]. Miklos et al. studied the different types of AOP including ozonation. Ozonation of CECs in aqueous solutions is achieved through two ways: direct reaction with molecular ozone (O_3_), or indirect reaction with free radicals (·OH) [73]. This technique has been used in wastewater treatment achieving positive effects for water disinfection, reduction of organic matter, and degradation of refractory contaminants [39,74,75]. Thanks to its high oxidation potential, ozonation facilitates disinfection, discoloration, and taste and odor control in drinking water and wastewater treatment [76,77]. ·OH formation is the main route for reducing pharmaceuticals such as CBZ present in wastewater. O_3_ radicals also favor the reduction but to a lesser extent than the ·OH [78,79].

There have been a great number of investigations into the reduction of pharmaceuticals in water, based on the combination of oxidation treatment together with adsorption treatment (O_3_/PAC). Chedeville et al. studied the reduction of fluoxetine and metoprolol [80]. Nebout et al. studied the reduction of metoprolol, ketoprofen, CBZ, terbutaline, fluoxetine, and sulfamethoxazole [81]. Beltrán et al. and Rozas et al. studied the reduction of DCF [82,83]. Comninellis et al. and Nguyen et al. highlighted that combined treatments improved CBZ reduction, although membrane filtration and MBR treatments were the most popular among the combined systems [84,85].

However, all these investigations are based on the combined treatment as a whole; no study references were found based on our experimental conditions because the adsorption and oxidation treatments in this investigation are carried out in phases and not together. The purpose of this work is to study the effect that the treatment with O_3_ followed by activated carbon or vice versa has on the time required for the removal of DCF and CBZ to reach eliminations higher than 90%, when compared with individual treatments. For this purpose, the time required to achieve these percentages in single and combined treatments was studied. The study was performed for two initial contaminant concentrations: 0.7 mg L^−1^ and 1.8 mg L^−1^. The ozone doses used were between 0.056 and 0.280 mg L^−1^ and the activated carbon doses were between 4 mg L^−1^ and 20 mg L^−1^.

## 2. Materials and Methods

### 2.1. Reagents

The CBZ (CAS No: 298-46-4) and DCF (CAS number: 15307-86-5) standards were supplied by the manufacturer Sigma-Aldrich (purity of 98–99%). The standards were diluted with methanol to concentrations of 2500 mg L^−1^. Final studied dilutions of 0.7 and 1.8 mg L^−1^ were obtained by dilution with acetonitrile and pure water with a ratio *v*:*v* = 55:45 with 1 mL of ortho-phosphoric acid (85% purity) for CBZ and *v*:*v* = 65:35 with 1 mL of ortho-phosphoric acid for DCF. Table 1 summarizes the physicochemical characteristics of the compounds studied.

### 2.2. Analytical Method

CBZ and DCF concentration were determined using high-performance liquid chromatography (HPLC) equipment from Agilent technologies (Agilent 1100 Series) (Bad Homburg v. d. Höhe, Germany). As stationary phase, a 5 µm Ascentis RP-Amide column, with a length of 150 mm and a diameter of 4.6 mm (Sigma-Aldrich) was used. The ultraviolet detector worked at a wavelength of 220 nm for CBZ and 286 nm for DCF. The mobile phase consisted of a mixture of acetonitrile and water (*v*:*v* = 55:45 for CBZ and *v*:*v* = 65:35 for DCF) with 1 mL of ortho-phosphoric acid, at a flow of 1.0 mL min^−1^ with a detection time of 180 s for CBZ and 210 s for DCF.

Studies by several authors [86,87,88] for direct reactions of ozone with several drinking water contaminants noted that acetonitrile can affect radical processes by scavenging hydroxyl radicals, albeit relatively slowly (k = 2.2 × 10^7^ M^−1^ s^−1^).

The linearity of the calibration curves was verified using standard solutions in the concentration range of 0.01–5.0 mg L^−1^; linear R-square values higher than 0.999 were obtained. Each standard was injected in triplicate. The limits of detection (LOD) and limits of quantification (LOQ) of the pharmaceutical products were: LOD and LOQ of CBZ were 0.02 mg L^−1^ and 0.06 mg L^−1^ and for DCF the values were 0.01 mg L^−1^ for LOD and 0.03 mg L^−1^ for LOQ.

### 2.3. Adsorption Tests

Adsorption tests were performed according to ASTM standard [89]. Each experiment was carried out in 0.5 L borosilicate amber glass bottles with lined screw caps in a stirred reactor with a fixed agitation speed of 300 rpm ± 10 rpm. The experiments were carried out at a temperature of 25 °C ± 1 °C. The variables studied were concentration of CBZ and DCF, 0.7 and 1.8 mg L^−1^ (± 0.1 mg L^−1^), and PAC dose between 1–20 mg L^−1^. The adsorption experiments were carried out for 24 h. During the adsorption test, samples were taken and once filtered (PVDF filter 0.22 µm) they were subjected to analysis. The filter reduced the initial concentration between 0.030 and 0.050 mg L^−1^ for both drugs.

### 2.4. Oxidation Tests

Ozone was generated using the Anseros COM-AD-01 equipment (Tubinga, Germany). The equipment was connected to an industrial oxygen supply with a purity of 99.5%, H_2_O 30 vpm, and a constant flow rate of 100 L h^−1^ at a pressure of 1 bar. A reactor volume of 0.5 L (borosilicate amber glass bottles with lined screw caps) was used; a constant stirring speed of 300 rpm ± 10 rpm and a temperature of 25 °C ± 1 °C were maintained. The variables studied were contaminant concentration (0.7 and 1.8 mg L^−1^) and ozone dose (0.056–0.280 mg L^−1^). The ozone doses studied were introduced continuously in each test. The maximum experimentation time was 20 min. During the test time, samples were extracted and analyzed to determine their concentration. The sampling was carried out manually using a syringe at different time intervals. The content of the syringe was quickly transferred to initially prepared vials containing 100 μL of 0.1 M sodium thiosulphate (Na_2_S_2_O_3_) solution to quench any remaining aqueous ozone in the solution [90].

### 2.5. Combined Tests

The O_3_ treatment was followed by PAC or vice versa (PAC followed by O_3_). In both cases the first treatment was maintained until a 50% reduction of the contaminant was achieved and then, the second phase was carried out. In the second phase, the trial was continued until the percentage of pharmaceutical elimination was at least 90%. In all these experiments, an ozone dose of 0.224 mg L^−1^, and a PAC dose of 16 mg L^−1^ were always used. The experiments were performed for an initial DCF and CBZ concentration of 0.7 and 1.8 mg L^−1^. The same experimental model indicated in Section 2.3 and Section 2.4 was used in each phase.

## 3. Results and Discussion

### 3.1. Activated Carbon

**Figure 1:**
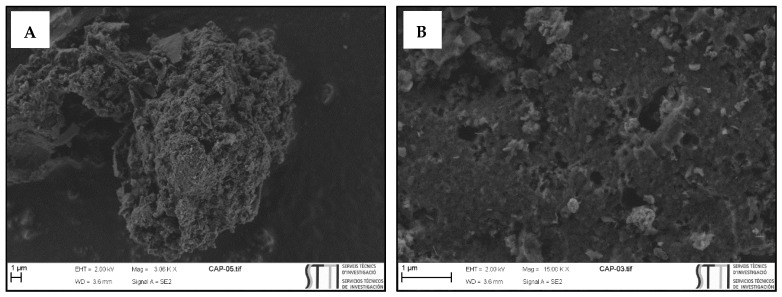
PULSORB PWX-HA images with scanning electron microscopy: (**A**) Mag = 3.06 K X; (**B**) Mag = 15.00 K X.

Activated Carbon PULSORB PWX-HA was used in this study (Figure 1). The carbon was characterized using the scanning electron microscopy (SEM) technique and the N_2_ physical adsorption technique at 77K. Based on analysis, it was determined that the activated carbon used in this study had the following characteristics: the specific surface area of PAC (based on BET) was 824.688 m^2^ g^−1^ and the average diameter of PAC particles was 1.180 nm. The number of pores greater than 2 nm was 63% and less than 2 nm of diameter was 37%. From the study of N2 adsorption isotherm, and taking into account IUPAC classification [91], the shape of the isotherm would correspond to type I, which is characteristic of solids with a micropore structure. The SEM technique enabled high resolution images of the studied activated carbon and its chemical composition. The structure was formed by 85% of carbon followed by 11% of oxygen due to the presence of various functional groups such as carboxylic acids, phenolic groups, or carbonyls.

### 3.2. Carbamazepine and Diclofenac Adsorption Tests

**Figure 2:**
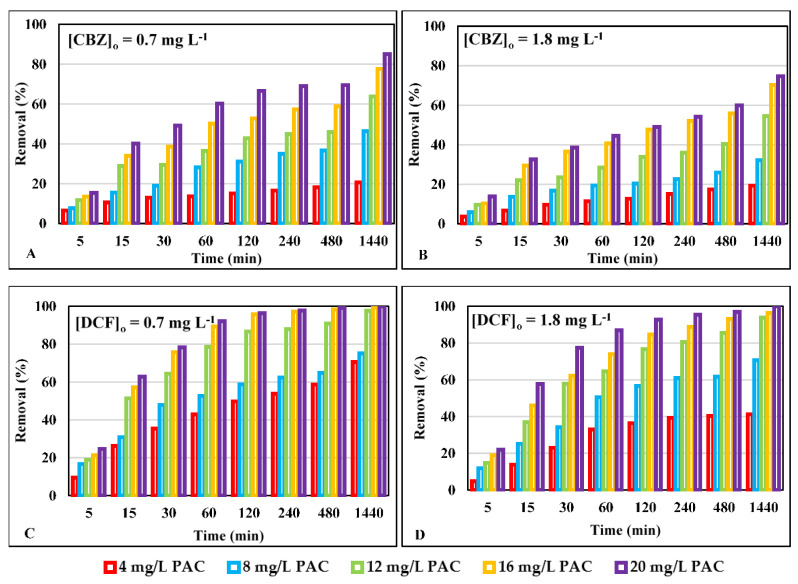
Removal percentages of CBZ and DCF over time at different PAC doses (4–20 mg L^−1^): (**A**) [CBZ]_0_ = 0.7 mg L^−1^; (**B**) [CBZ]_0_ = 1.8 mg L^−1^; (**C**) [DCF]_0_ = 0.7 mg L^−1^; (**D**) [DCF]_0_ = 1.8 mg L^−1^.

Figure 2 shows the percentage removal of CBZ and DCF concentration over time when it was put in contact with different doses of PAC between 4 and 20 mg L^−1^. Figure 2A,C refer to an initial CBZ or DCF concentration of 0.7 mg L^−1^. Figure 2B,D refer to an initial concentration of 1.8 mg L^−1^.

For both contaminants, an increase in carbon dosage and contact time allows the contaminants to bind more to the surface of the activated carbon, due to an increase in the area where they can be adsorbed. However, there must be a balance between the dose of activated carbon and the required contact time in order to make the process economically viable as much as possible. In the case of CBZ, for any of the concentrations studied or doses of activated carbon used, elimination percentages higher than 85% were not reached. In the case of DCF for the highest concentration of contaminant studied, after 120 min and with the two highest doses of carbon used, the value of 90% contaminant elimination was exceeded.

As can be seen from these graphs, the removal of DCF is in all cases superior to that of CBZ. For example, for a contact time of 30 min at a dose of 12 mg L^−1^ of carbon, at the lowest contaminant dose studied (0.7 mg L^−1^), we achieved DCF reductions greater than 60%, while for CBZ it does not exceed 29%. At a contact time of one hour, with a dose of 16 mg L^−1^ of PAC, the percentage of DCF removal is 90% while for CBZ it is 50%. In all cases, an increase in the carbon dose improves contaminant removal. However, after 60 min and for the contaminant that is most removed (DCF), an increase between 16 and 20 mg L^−1^ of carbon does not significantly improve the results.

The size of CBZ (0.84 nm) and DCF (0.93 nm) molecules (Table 1) is smaller than the average pore size of PAC. Therefore, the accessibility of the molecules to the activated centers of the activated carbon could occur; however, due to the size of the molecule it would be expected that CBZ would be more adsorbed due to its greater accessibility, but this is not the case. There are different reasons why the size of the molecule is not the only determining factor [92,93]. There are different reasons for the greater adsorption of DCF compared with CBZ. Some authors such as Contreras et al. indicate that the presence of electro-attracting groups, such as F, Cl, and Br increase the affinity of the adsorbate on the activated carbon because they decrease the electronic density in the aromatic ring [94]. In this case, DCF has two chlorine groups attached to one of the aromatic rings, thus enhancing its adsorption on carbon.

Moreover, other authors such as Aylas Orejón et al. suggest that the adsorption of DCF is also favored by its higher molecular weight compared with CBZ (296 for DCF and 236 for CBZ) due to the effect of London molecular interactions, which are directly proportional to the molar mass [95]. It is also important to know the dissociation constant (pKa) of each adsorbate. In the case of DCF with a carboxylic acid group, it is 50% ionized at pH 4.15 and entirely ionized at pH 7. On the other hand, the amine group of CBZ is neutral (NH_2_) at pH 7 and 50% ionized (negative charge) at pH 13.9. In general, compounds with a high pKa value tend to remain in water in their ionic form; therefore, their adsorption decreases, which would also justify that CBZ is adsorbed in a lower percentage than DCF.

Hydrophobic interactions is another important factor, according to Nam et al. hydrophobic interaction between the contaminant and activated carbon is one of the primary adsorption mechanisms [96]. DCF has a Log Kow value 4.51 higher than CBZ (2.3). This means that DCF is more hydrophobic than CBZ, which favors the higher adsorption of DCF.

These results are in agreement with other investigations on the reduction of CBZ in water by adsorption with PAC [97,98,99]. Meinel et al. studied the reduction of CBZ with an initial concentration of 0.89 µg L^−1^ and PAC doses of 10 and 30 mg L^−1^ with a contact time of 30 min, reaching a reduction of 39% and 65%, respectively [100]. Margot et al. and Altmann et al. obtained reductions greater than 90% with PAC doses of 13 and 20 mg L^−1^ with 30 min of contact for an initial concentration of CBZ of 2.5 μg L^−1^ [101,102].

The results obtained in this research also confirm those obtained by other authors such as Margot et al. and Altmann et al. where they achieved reductions greater than 90% with PAC doses of 13 and 20 mg L^−1^ with 30 min of contact for initial concentrations of contaminant 1000 times lower than those of the present study [101,102]. Boehler et al. and Kovalova et al. obtained similar reductions with carbon doses of 15 mg L^−1^ and 23 mg L^−1^, respectively [97,98]. On the other hand, Stoquart et al. found that with an initial concentration of DCF 0.2 μg L^−1^ and a carbon dose of 1 g L^−1^, they obtained a reduction of 95% in less than 5 min [103]. Apopei et al. used an initial DCF concentration of 20 mg L^−1^ and a PAC dose of 1 g L^−1^, obtained a reduction of over 90% in the first minute and after 20 min the reduction reached was 99% [104]. Comparing the results obtained from other studies, it is observed that PAC has a high enough adsorption capacity to be able to retain higher concentrations of DCF than the usual ones in water and with similar doses, to achieve reductions in an optimal time.

The Langmuir and Freundlich adsorption models were studied with the aim of correlating the experimental data obtained. In addition, the kinetics of the process were studied by pseudo-first-order and pseudo-second-order models. For this purpose, the linearized equations of these models, which are widely used, were applied [105,106,107,108].

From the two adsorption isotherms studied (Langmuir and Freundlich) (Table 2) and considering the comparison of the R^2^ values, it was observed that the Freundlich model is better adjusted, considering a favorable adsorption process for both cases because its value of n is greater than 1. This type of isotherm indicates that there are active sites that have a heterogeneous distribution of adsorption energy that usually form multilayers of the molecules adsorbing on the PAC. The values of K_F_ (Freundlich constant) are related to the binding energy and adsorption capacity being higher for DCF than for CBZ. The adsorption behavior for the two compounds follows pseudo-second-order kinetics.

Table 3 shows the results of the application of the pseudo-first-order and pseudo-second-order kinetic models. Considering the R^2^ value closer to 1, it was found that the pseudo-second-order kinetic model is the one that best describes the process. This corresponds to a process governed by chemisorption. The value of q_e_ increased with the initial concentration of the contaminant, although the percentage removal decreased with increasing initial concentration. As the concentration of contaminant increases, there is a decrease in the adsorption resistance and therefore the value of q_e_ increases due to the increase in driving force. The value of k_2_ (pseudo-second-order rate constant) was higher in DCF than in CBZ.

### 3.3. Carbamazepine and Diclofenac Oxidation Tests

**Figure 3:**
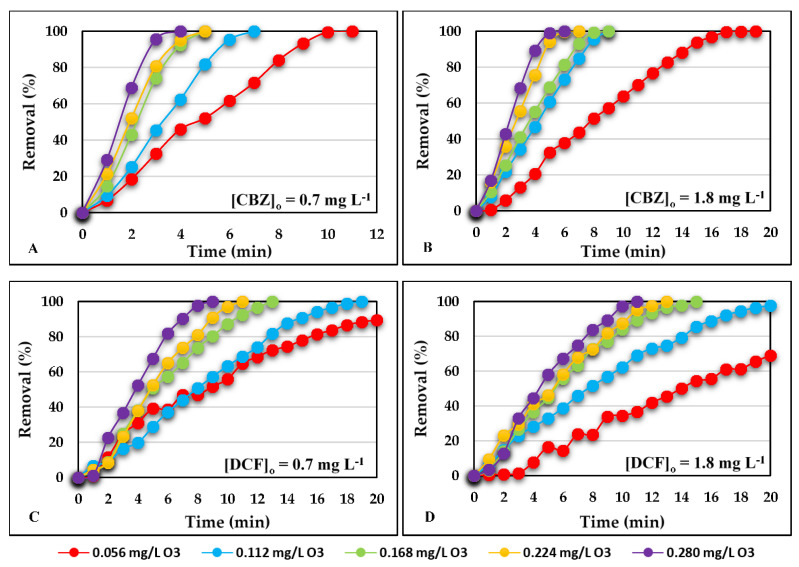
Removal percentages of CBZ and DCF over time at different ozone 0.056 mg L^−1^–0.280 mg L^−1^: (**A**) [CBZ]_0_ = 0.7 mg L^−1^; (**B**) [CBZ]_0_ = 1.8 mg L^−1^; (**C**) [DCF]_0_ = 0.7 mg L^−1^; (**D**) [DCF]_0_ = 1.8 mg L^−1^.

Figure 3 shows the percentage removal of CBZ and DCF over time for the different ozone doses studied. Figure 3A,C refer to an initial CBZ or DCF concentration of 0.7 mg L^−1^. Figure 3B,D refer to an initial concentration of 1.8 mg L^−1^.

For all the contaminant concentrations studied and at any ozone dose applied, the removal of CBZ was higher than the removal of DCF. The higher the doses of ozone, the greater the contaminant removal, although these differences are less significant at doses of 0.224 mg L^−1^ of ozone and higher.

For the lowest contaminant concentration studied and at the lowest ozone dose applied, a reduction of CBZ greater than 90% was achieved after 9 min, while the time required for DCF reduction to reach 90% was 20 min.

When an ozone dose of 0.280 mg L^−1^ was applied, the percentage of CBZ reduction was higher than 90% before reaching 3 min and when an initial contaminant concentration of 1.8 mg L^−1^ was used, this percentage of reduction was reached at 4 min. In the case of DCF, for the same ozone dose, the times were 7 min and 9.5 min for the two initial concentrations studied, respectively.

There are multiple studies based on the oxidation of CBZ that mention the effectiveness of ozone treatment in reducing this pharmaceutical [102,109,110,111]. However, it must be taken into account that most of the research is based on wastewater from different WWTPs, which present various factors that interact in the reduction of CBZ, such as organic matter and other substances present in the water [112].

Alharbi et Price indicated that drug reduction by O_3_ and ·OH oxidation treatment is very effective [79]. They observed that for an initial concentration of 5 mg L^−1^, reductions greater than 99% were achieved when they used an ozone dose of 1.6 mg L^−1^ for CBZ and 2.3 mg L^−1^ for DCF. These results are in agreement with those obtained in the present investigation, where CBZ removal is superior to that of DCF.

Andreozzi et al. achieved a CBZ reduction of greater than 99% when they treated an initial concentration of 0.8 mg L^−1^ and with an O_3_ dose of 1 mg L^−1^ in less than 5 min [113]. Rozas et al., using an initial CBZ concentration of 2.8 mg L^−1^ and an O_3_ dose of 1.8 mg L^−1^, also achieved a reduction of more than 99% at 3.5 min [83]. Although the initial concentration and O_3_ dose were higher than those of this study, it shows the great effectiveness of the treatment in reducing CBZ of concentrations higher than those found in the effluents of WWTPs. However, in research by Justo et al. for a CBZ initial concentration of 1038 µg L^−1^, they achieved a reduction of 32% with a dose of 0.14 mg O_3_ mg TOC^−1^, and when increasing the dose to 2.78 mg O_3_ mg TOC^−1^ the percentage rose to 99% [114].

Mcdowell et al. studied the process of CBZ ozonation and observed that O_3_ reacts rapidly with the double bond, giving rise to several by-products that contain functional groups based on quinazoline [115]. Among these by-products they detected 1-(2-benzaldehyde)-4-hydro-(1H, 3H)-quinazoline-2-one (BQM), 1-(2-benzaldehyde)-(1H, 3H)-quinazoline-2,4-dione (BQD), and 1-(2-benzoicacid)-(1H, 3H)-quinazoline-2,4-dione (BaQD). Hübner et al. identified up to 13 by-products, establishing that the reaction follows the Criegee mechanism, with the stoichiometric formation of BQM as the primary product and BQD and BaQD as a secondary reaction with O_3_. Therefore, as the main contaminant is reduced, new chemical species emerge [116].

Various studies also confirm the effectiveness of oxidation treatment with high doses of O_3_ for the reduction of DCF at low concentrations [102,117]. Rozas et al. studied the reduction of DCF with an initial concentration of 2.8 mg L^−1^ in an ultra-pure water matrix and O_3_ dose of 1.8 mg L^−1^ [83]. In that study, DCF reductions of greater than 99% were achieved at 3.5 min. In our study, although the initial concentration of the contaminant was lower, we obtained a complete reduction of DCF with lower doses of O_3_. This ease of reduction is due to the large number of electrons in the functional groups, facilitating the reduction of DCF even at low doses of O_3_ [118], with a kinetic constant (kO_3_) of high oxidation (kO_3_ > 10^4^ M^−1^ s^−1^) [119,120,121]. Justo et al. studied the reduction of DCF with an initial concentration of 0.605 µg L^−1^, obtaining a reduction of 57% with an ozone dose of 0.14 mg O_3_ mg TOC^−1^ [114]. In that study, increasing the dose to 2.78 mg O_3_ mg TOC^−1^ achieved a reduction of 99%.

Alharbi et al. studied the by-products that were generated during the ozonation treatment of DCF. Their study indicates that by-products can be generated in two ways: (1) directly—O_3_ attacks the electrophilic positions in the aromatic ring, and electron donor groups such as hydroxyl and amine groups induce a high electron density in the ortho and para positions; and (2) indirectly—the ·OH generated in the decomposition of O_3_ attacks the positions of the most susceptible molecule, making it possible to form positional isomers in the aqueous solution [122]. In other studies, by-products of DCF ozonation were detected, including 2-[2,6-dichlorophenyl)-4-hydroxyphenyl) amino]-phenylacetic acid and 2-[2,6-dichlorophenyl) amino]-5-hydroxyphenylacetic acid [123,124]. Coelho et al. studied the ozonation process of DCF, proposing a degradation route following different routes. In this study, the generation of 18 by-products was described [124]. Some of these by-products have also been reported by other authors with other treatments used such as: Photo-Fenton, heterogeneous catalytic oxidation, photolysis, and solar degradation [125,126,127,128,129].

When ozonation experiments were carried out for both drugs, the solution reached for the lowest dose of O_3_ a pH value of around 5.0, and a pH value of 4.3 for the highest dose of O_3_. At acid pH, the generation of ·OH (E_0_ = 2.8 eV) is lower to generation of O_3_ (E_0_ = 2.07 eV) [130].

The lower reduction obtained by DCF compared with CBZ in oxidation treatments is mainly due to the chemical structure of these contaminants. Both drugs have main compounds in common (C, N, O, H), but DCF has a structure formed by an amine located between two benzene rings (one with two chlorine atoms and another with a carboxyl group) (see Table 1). The presence of these chlorine groups in the benzene ring of the DCF molecule causes a decrease in the density of electrons, decreasing the electrophilic attack of ozone [131]. When O_3_ reacts with DCF, it oxidizes the secondary amine, leaving the compound with a double bond, with this double bond being more resistant to oxidation.

CBZ presents kinetic constant values of high oxidation in the C = C of the molecule [102,121], and high reduction percentages due to the large number of electrons in the functional groups, facilitating an easy reduction in effluents, even at low doses of O_3_ [118]. The majority of researches are based on wastewater matrices from different WWTPs analyzing various factors such as the variation in natural organic matter (NOM) of the effluent or the quality of water [112]. When real wastewater or surface water was used, the presence of NOM also competes with organic contaminants for oxidants and thus decreases their eliminations in water matrices [132]. Humic acid had negative influence on the degradation efficiency of DCF, which decreased from 80.8% to 62.9% when humic acid concentration was 30 mg L^−1^, mainly due to the competition between humic acid and DCF for OH radicals [133].

### 3.4. Combined Tests for Carbamazepine and Diclofenac PAC/O_3_ and O_3_/PAC

**Figure 4:**
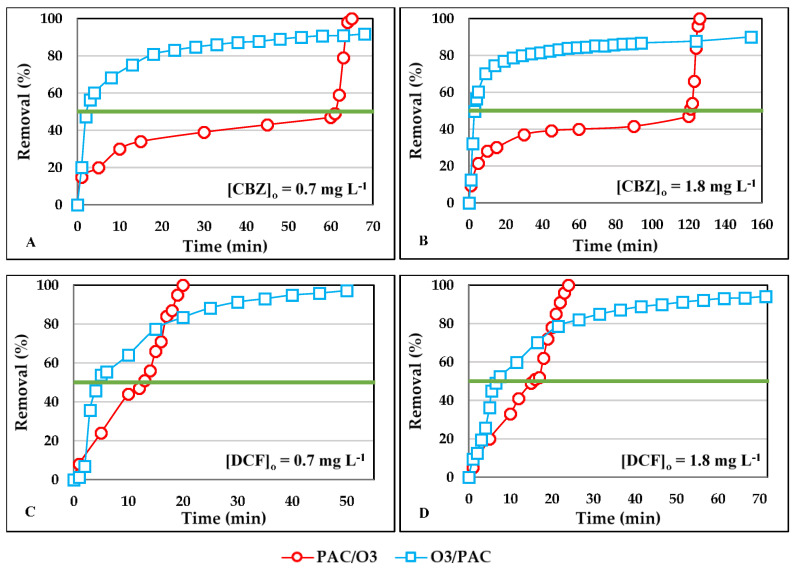
Removal percentages of CBZ and DCF over time for combined treatments O_3_/PAC and PAC/O_3_. PAC doses = 16 mg L^−1^ and O_3_ doses = 0.224 mg L^−1^: (**A**) [CBZ]_0_ = 0.7 mg L^−1^; (**B**) [CBZ]_0_ = 1.8 mg L^−1^; (**C**) [DCF]_0_ = 0.7 mg L^−1^; (**D**) [DCF]_0_ = 1.8 mg L^−1^.

The application of O_3_ followed by PAC and vice versa was studied. All studies were conducted for a PAC dose of 16 mg L^−1^ and an O_3_ dose of 0.224 mg L^−1^. Figure 4 shows the removal percentage achieved over time for the two contaminants studied. The first treatment was carried out until the contaminant reduction was 50%, then the second treatment was applied to reduce the contaminant to above 90%. Figure 4A,C refer to an initial CBZ or DCF concentration of 0.7 mg L^−1^. Figure 4B,D refer to an initial concentration of 1.8 mg L^−1^.

For CBZ, at either of the two concentrations studied, in general O_3_/PAC treatment obtained higher elimination percentages in less time than when PAC/O_3_ was used. The time required was also shorter than when only activated carbon was used but it was longer than when only ozone was used. For example, to obtain a reduction of 90% of CBZ when we treated an initial concentration of 0.7 mg L^−1^, it took more than 1440 min to reach that value when PAC was used, 4 min using O_3_, 63 min when PAC/O_3_ was used, and 52 min using O_3_/PAC. To obtain yields above 90%, the PAC treatment followed by O_3_ was faster. For the initial CBZ concentration of 1.8 mg L^−1^, the times were over 1440 min with PAC, 5 min with O_3_, 125 min with PAC/O_3_, and 154 min with O_3_/PAC. However, for values below 90%, in all cases the combined O_3_/PAC treatment achieved high removal efficiencies in less time than PAC/O_3_, with the advantage of removing the by-products produced during oxidation.

When ozone reacts with pharmaceutical products, in addition to reducing CBZ, it also generates by-products in this reaction. Therefore, when the combination of O_3_/PAC was carried out, at the end of the first treatment with O_3_, the CBZ that was not reduced in this first process was still present in the water but with its by-products; when the second treatment with activated carbon began, it adsorbed the CBZ and the by-products. However, when the combination was PAC/O_3_, in the second treatment, the ozone reduced the CBZ present in water but it generated new chemical species with negative effects on the aquatic environment.

Other research based on the combination of various treatments for the reduction of CBZ were based mainly on the combined treatments of AC with membranes: PAC/MBR [134], MBR/PAC [135], and PAC/MF [136]. In these studies, reductions between 10% and 20% were obtained depending on the treatment and which membranes were used. These percentages increased up to 90% with the incorporation of PAC doses of 1 g L^−1^. In a study carried out by Im et al. based on the combination of AOP (O_3_/UV/H_2_O_2_), the complete reduction of CBZ was mostly dependent on the dose of ozone used [137].

In the case of DCF, we observed that in Figure 4 the data of the two treatments cross at a yield of 80%. At this point, PAC/O_3_ and O_3_/PAC obtain the same yield in the same time (16 min and 20 min for initial DCF concentrations of 0.7 and 1.8 mg L^−1^, respectively), but for percentages lower than this point the fastest treatment is O_3_/PAC, while for higher yields the fastest treatment is PAC/O_3_. When an initial DCF concentration of 0.7 mg L^−1^ was treated, the time required to achieve 90% contaminant reduction was 60 min with PAC, 10 min with O_3_, 19 min with PAC/O_3_, and 30 min with O_3_/PAC. When the initial concentration of DCF was 1.8 mg L^−1^, the treatment times were 240, 10, 22, and 47 min when treated with PAC, O_3_, PAC/O_3_, and O_3_/PAC. Furthermore, the O_3_/PAC treatment would reduce the presence of oxidation by-products.

With an initial DCF concentration of 30 mg L^−1^ and a dose of 20 mg L^−1^ of O_3_, Beltrán et al. observed that with only the O_3_ treatment, they were unable to reduce the compounds formed during the first minutes of ozonation [82]. Most of the products detected were carboxylic acid, since they are refractory under attack by O_3_. In contrast with the combined O_3_/AC treatment, using a carbon dose of 20 g L^−1^, reductions of over 99% were achieved in about 10 min. It was also observed that the longer the reaction time of the O_3_, the lower the affinity of the DCF by-products to be adsorbed on the surface of the AC. Rozas et al. studied the reduction of DCF through the combination of O_3_/AC [83]. They noticed that with the O_3_ treatment they achieved a greater reduction of the DCF in less time than with the AC adsorption treatment. This fact corroborated research by Wang et al. which found that with the treatment of O_3_, reductions of more than 99% of DCF were achieved in various water matrices, without the need to combine treatments since the reduction of pharmaceuticals is not improved and the cost increases [138].

No studies were found based on the combination of AC adsorption treatments followed by ozonation or vice versa. The vast majority of studies about the reduction of DCF have been based on combination and not on phases.

In view of the results obtained, the hybrid treatments studied in this research (O_3_/PAC or PAC/O_3_) are effective for the removal of DCF and CBZ. However, the treatment starting with O_3_ followed by AC is recommended because in addition to reducing DCF or CBZ, the by-products formed during oxidation with ozone could probably be reduced (see Supplementary Material Figures S1–S4). This recommended treatment would benefit the ecological status of the waters and also the health of living organisms [139].

## 4. Conclusions

From our study it can be deduced that, in the case of CBZ, the combined treatment of O_3_/PAC significantly reduces the contact time to reach reduction percentages of up to 90%. In addition, it has the advantage that the final adsorption treatment could reduce the presence of oxidation by-products in the final effluent. It has been shown that to achieve a reduction of 90% of this contaminant, the PAC/O_3_ treatment would be faster, even though it would generate oxidation by-products.

With combined treatments, the reduction of DCF requires, in all cases, less treatment time than CBZ. In the case of DCF, its concentration is reduced by combined treatments in less time than CBZ due to the fact that the limiting process is adsorption and, as we have already seen in Section 3.1, DCF is adsorbed more effectively on PAC.

For a PAC dose of 16 mg L^−1^ and ozone dose of 0.224 mg L^−1^, to achieve a reduction of DCF greater than 90% using O_3_/PAC, the time required to treat a concentration of 0.7 and 1.8 mg L^−1^ was 30 min and 47 min, respectively, while the times to treat CBZ were 53 min and 154 min. This represents a substantially shorter contact time compared with the time required for a PAC only treatment.

This research therefore provides evidence of the important benefits that hybrid treatments have in terms of reducing the presence of drugs in water, especially in the case of CBZ. However, as it has been shown, it will be necessary to carry out specific studies for each contaminant because the differences between one and the other can be significant.

## Figures and Tables

**Table 1:** Physicochemical characteristics of CBZ and DCF [8].

CEC	Molecule Size (nm)	Molar Mass (g mol^−1^)	Solubility in Water (mg L^−1^)	Log Kow	pKa
CBZ 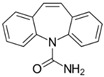	0.84	236.27	18.00 (25 °C)	2.3	13.9
DCF 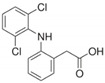	0.93	296.10	2.37 (25 °C)	4.51	4.15

**Table 2:** Freundlich and Langmuir Isotherm parameters for DCF and CBZ.

CEC (mg L^−1^)	Freundlich			Langmuir		
	K_F_ (L g^−1^)	n	R^2^	q_e_ (mg g^−1^)	b (L mg^−1^)	R^2^
DCF 0.7	192.24	4.42	0.998	89.08	827.02	0.817
DCF 1.8	167.13	4.06	0.990	144.32	56.09	0.811
CBZ 0.7	68.70	11.17	0.990	57.41	1254.14	0.846
CBZ 1.8	80.42	4.39	0.990	99.93	4.34	0.957

K_F_—Freundlich adsorption equilibrium constant; n—Freundlich intensity factor; q_e_—adsorption capacity at equilibrium time; b—constant related to the affinity between adsorbate and adsorbent of Langmuir’s model; R^2^—goodness-of-fit.

**Table 3:** Kinetic parameters for DCF and CBZ at different initial contaminant concentrations: pseudo-first-order, pseudo-second-order models.

CEC (mg L^−1^)	Pseudo-First-Order Model			Pseudo-Second-Order Model			
	q_e_ (mg g^−1^)	k_1_ 10^−3^ (min^−1^)	R^2^	q_e_ (mg g^−1^)	h (mg g^−1^ min^−1^)	k_2_ 10^−4^ (g mg^−1^ min^−1^)	R^2^
DCF 0.7	20.590	23.19	0.870	52.550	5.978	21.60	1.000
DCF 1.8	51.261	5.374	0.735	115.864	5.419	4.04	1.000
CBZ 0.7	29.615	2.118	0.932	47.162	0.588	2.64	0.997
CBZ 1.8	40.398	1.243	0.765	83.411	1.464	2.10	0.996

q_e_—amounts of adsorbed contaminants at equilibrium; k_1_—rate constant of pseudo-first-order adsorption; k_2_—rate constant of pseudo-second-order adsorption; h—initial adsorption rate; R^2^—goodness-of-fit.

## Supplementary Materials

The following are available online at https://www.mdpi.com/article/10.3390/ijerph18137163/s1. Figure S1: High performance liquid chromatography (HPLC) results combined test CBZ + O_3_ + PAC: (A) Initial DCF solution 0.7 mg L^−1^; (B) After O_3_ treatment (0.224 mg L^−1^ O_3_); (C) O_3_ + PAC (16 mg L^−1^); (D) Combined representation of A, B and C. Figure S2: High performance liquid chromatography (HPLC) results combined test CBZ + O_3_ + PAC: (A) Initial DCF solution 1.8 mg L^−1^; (B) After O_3_ treatment (0.224 mg L^−1^ O_3_); (C) O_3_ + PAC (16 mg L^−1^); (D) Combined representation of A, B and C. Figure S3: High performance liquid chromatography (HPLC) results combined test CBZ + O_3_ + PAC: (A) Initial CBZ solution 0.7 mg L^−1^; (B) After O_3_ treatment (0.224 mg L^−1^ O_3_); (C) O_3_ + PAC (16 mg L^−1^); (D) Combined representation of A, B and C. Figure S4: High performance liquid chromatography (HPLC) results combined test CBZ + O_3_ + PAC: (A) Initial CBZ solution 1.8 mg L^−1^; (B) After O_3_ treatment (0.224 mg L^−1^ O_3_); (C) O_3_ + PAC (16 mg L^−1^); (D) Combined representation of A, B and C.
