# Supplementary material for: Carbamazepine and Diclofenac Removal Double Treatment: Oxidation and Adsorption
Source: Int J Environ Res Public Health. 2021 Jul 4;18(13):7163. doi: 10.3390/ijerph18137163 (PMC8296929; doi:10.3390/ijerph18137163)
Supplement: Supplementary file 1 [file ijerph-18-07163-s001.zip › ijerph-1251683-supplementary.pdf]

SUPPLEMENTARY FILE

Figures 1-4 show the graphs of the HPLC detector response obtained from the different analyses for the combined O_3/PAC treatment at the initial concentrations of 0.7 and 1.8 mg L^{-1} for each of the compounds studied. For example, Figure 1A shows the chromatographic image from the analysis of the initial 0.7 mg L^{-1} diclofenac solution before treatment. Figure 1B shows the result after treatment with ozone, where it can be seen that the main peak decreases and at least two more peaks appear. Figure 1C shows the results when the solution, once treated with ozone, was subsequently treated with carbon. In Figure 1D all chromatograms are superimposed (A, B and c) on the same time line, we can see that the peaks that appeared in Figure 1B have decreased, so that the by-products generated in the ozonation when they were subsequently treated with PAC have been reduced. This behavior is similar for all the other compounds and initial concentrations.

1. Combined tests DCF 0.7 mg L^{-1}.

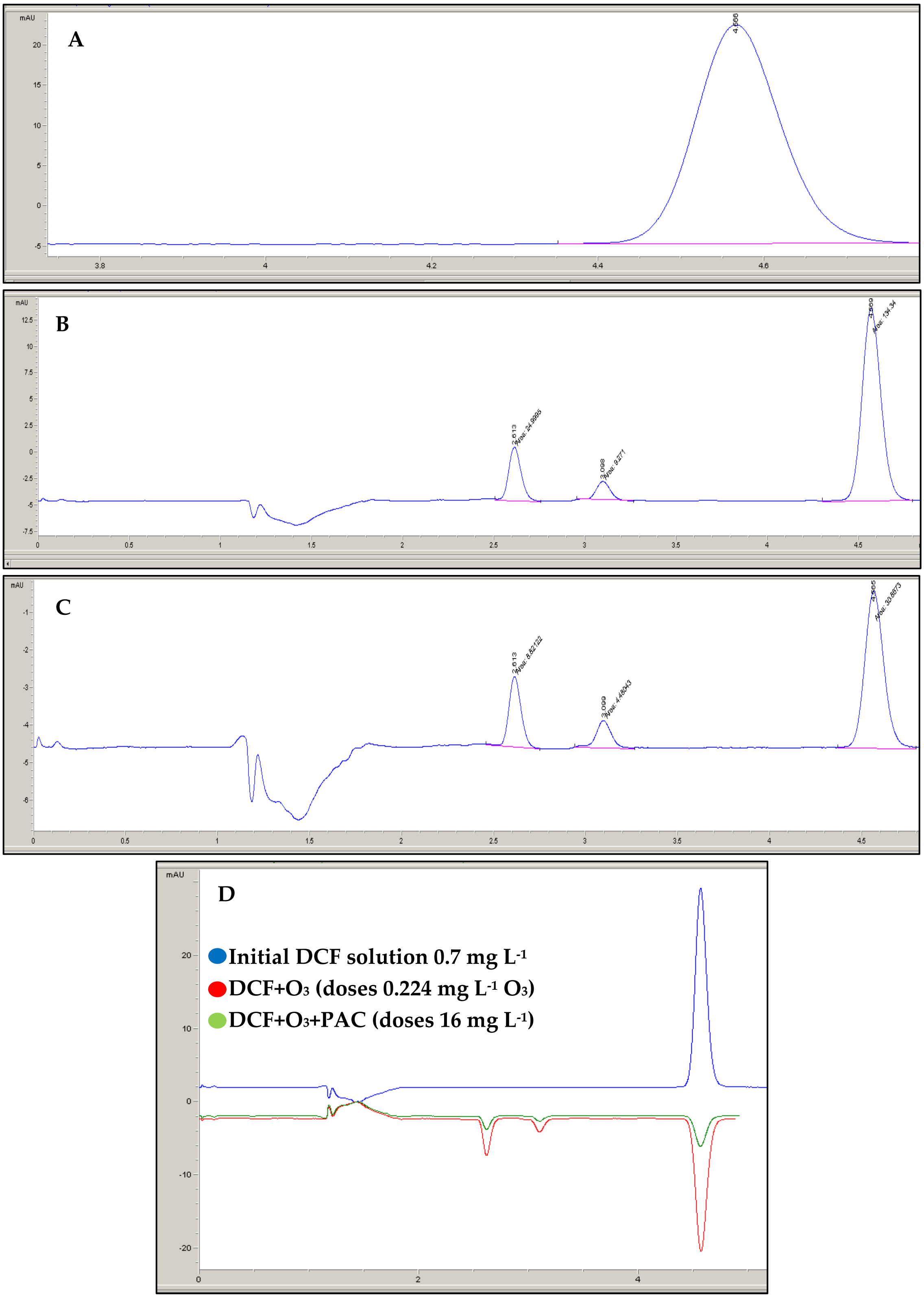

Figure 1S. High performance liquid chromatography (HPLC) results combined test CBZ+O_3+PAC: A) Initial DCF solution 0.7 mg L^{-1}; B) After O_3 treatment (0.224 mg L^{-1} O_3); C) O_3+PAC (16 mg L^{-1}); D) Combined representation of A, B and C.

2. Combined tests DCF 1.8 mg L^{-1}.

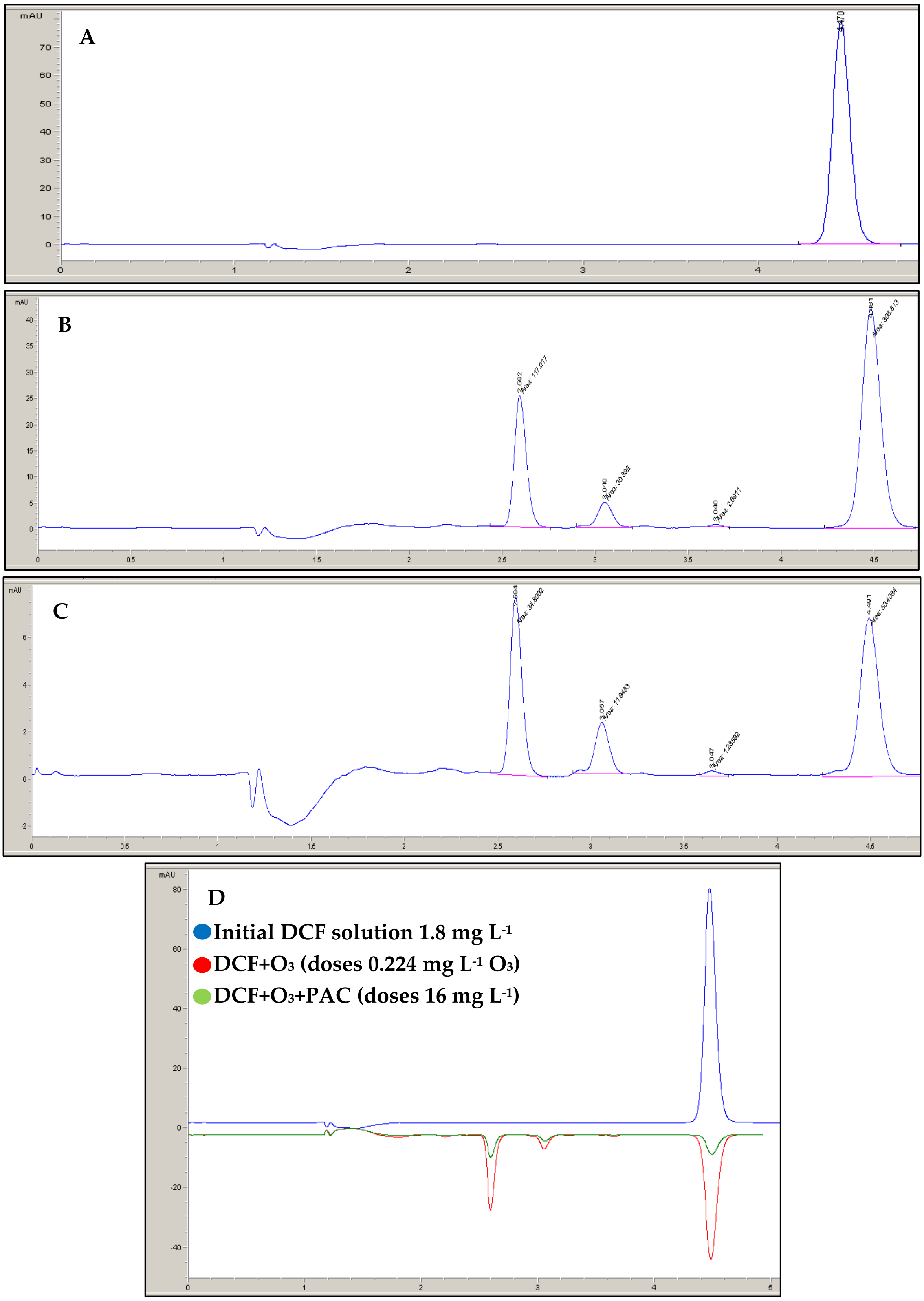

Figure 2S. High performance liquid chromatography (HPLC) results combined test CBZ+O_3+PAC: A) Initial DCF solution 1.8 mg L^{-1}; B) After O_3 treatment (0.224 mg L^{-1} O_3); C) O_3+PAC (16 mg L^{-1}); D) Combined representation of A, B and C.

3. Combined tests CBZ 0.7 mg L^{-1}.

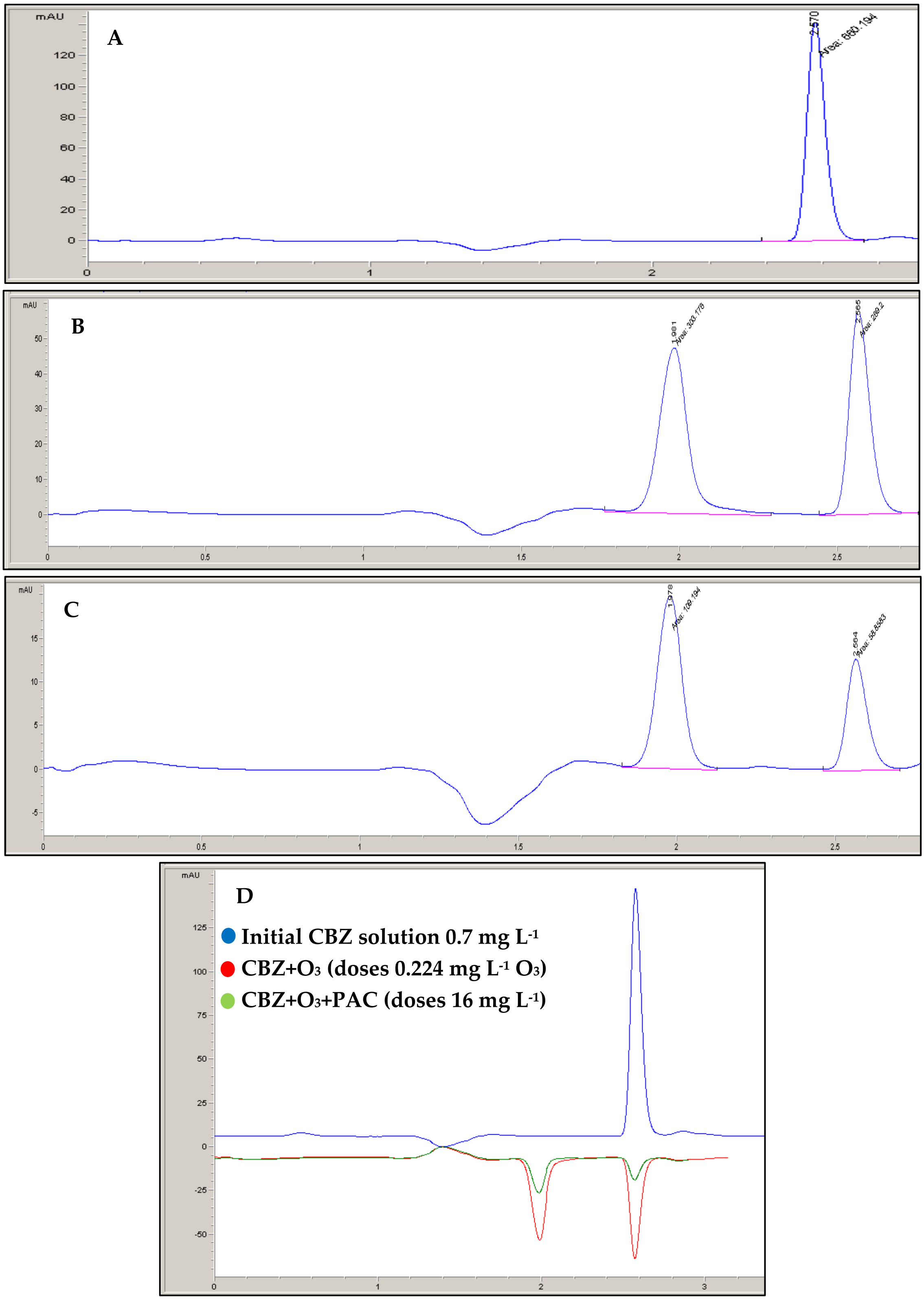

Figure 3S. High performance liquid chromatography (HPLC) results combined test CBZ+O_3+PAC: A) Initial CBZ solution 0.7 mg L^{-1}; B) After O_3 treatment (0.224 mg L^{-1} O_3); C) O_3+PAC (16 mg L^{-1}); D) Combined representation of A, B and C.

4. Combined tests CBZ 1.8 mg L^{-1}.

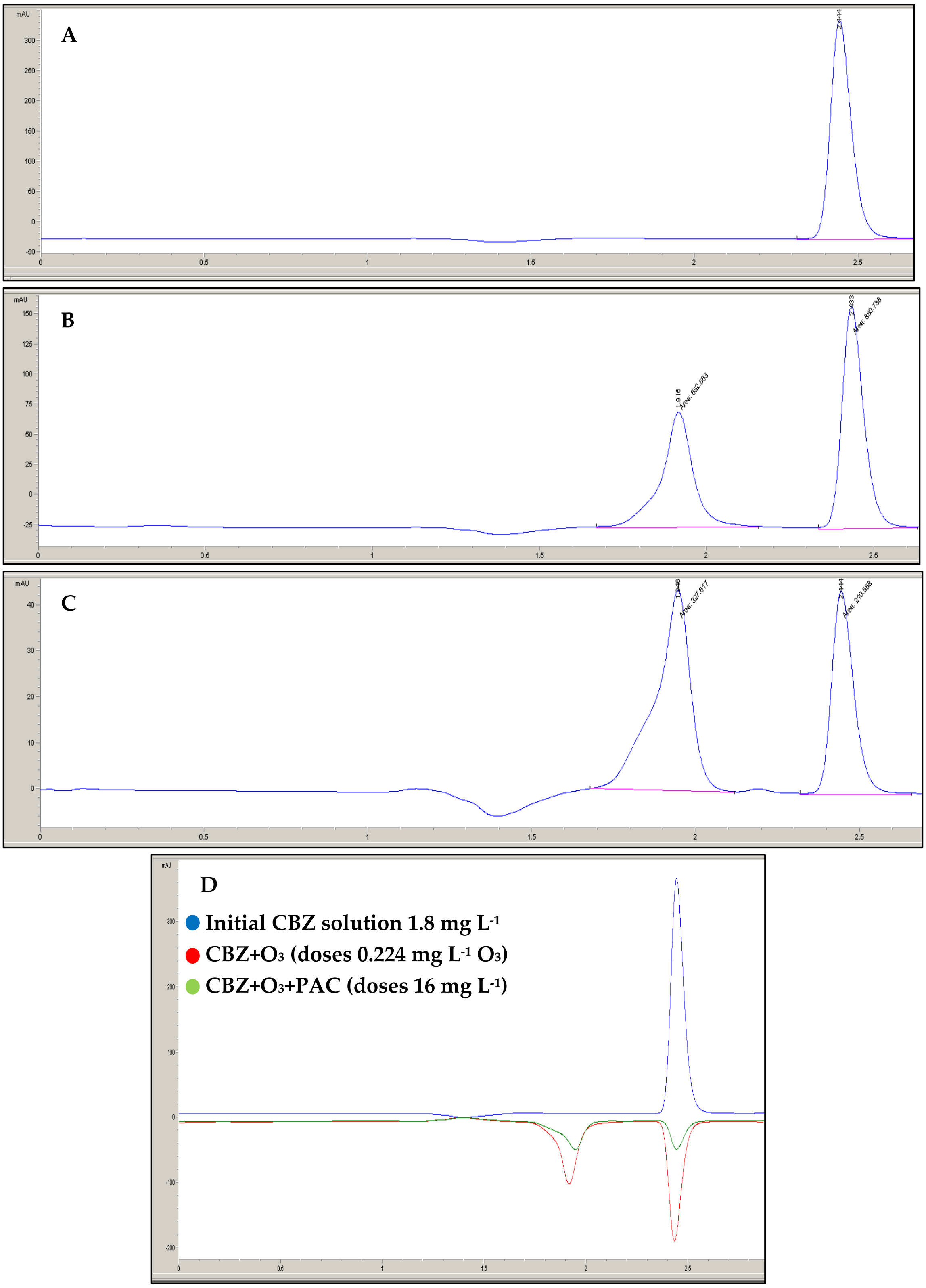

Figure 4S. High performance liquid chromatography (HPLC) results combined test CBZ+O_3+PAC: A) Initial CBZ solution 1.8 mg L^{-1}; B) After O_3 treatment (0.224 mg L^{-1} O_3); C) O_3+PAC (16 mg L^{-1}); D) Combined representation of A, B and C.
